# Muscle stem cell dysfunction impairs muscle regeneration in a mouse model of Down syndrome

**DOI:** 10.1038/s41598-018-22342-5

**Published:** 2018-03-09

**Authors:** Bradley Pawlikowski, Nicole Dalla Betta, Tiffany Elston, Darian A. Williams, Bradley B. Olwin

**Affiliations:** 1 Department of Molecular, Cellular and Developmental Biology, University of Colorado, 347 UCB, Boulder, CO 80309 United States; 2 Linda Crnic Institute for Down Syndrome, University of Colorado School of Medicine, Aurora, United States

## Abstract

Down syndrome, caused by trisomy 21, is characterized by a variety of medical conditions including intellectual impairments, cardiovascular defects, blood cell disorders and pre-mature aging phenotypes. Several somatic stem cell populations are dysfunctional in Down syndrome and their deficiencies may contribute to multiple Down syndrome phenotypes. Down syndrome is associated with muscle weakness but skeletal muscle stem cells or satellite cells in Down syndrome have not been investigated. We find that a failure in satellite cell expansion impairs muscle regeneration in the Ts65Dn mouse model of Down syndrome. Ts65Dn satellite cells accumulate DNA damage and over express Usp16, a histone de-ubiquitinating enzyme that regulates the DNA damage response. Impairment of satellite cell function, which further declines as Ts65Dn mice age, underscores stem cell deficiencies as an important contributor to Down syndrome pathologies.

## Introduction

Trisomy 21, responsible for Down syndrome, is the most common autosomal aneuploidy and the most frequent genetic cause of intellectual disability^1–3^. Cognitive disabilities, growth defects, muscle weakness, facial abnormalities, cardiac malformations, early-onset Alzheimer’s disease and premature aging manifest in Down syndrome with variable penetrance^4,5^. Although the cellular and molecular mechanisms driving these different phenotypes are incompletely understood, altered stem cell function is a potential common link. For example, expansion and differentiation defects in neuronal stem cells impair neurogenesis in the developing brain and adult brain of individuals with Down syndrome^6–8^. Hematopoietic stem cells accumulate DNA damage, prematurely senesce and fail to expand in mouse models of Down syndrome^9,10^. Thus, stem cell defects in Down syndrome likely contribute to cognitive impairments, blood cell disorders, and pre-mature aging phenotypes in Down syndrome^10–13^.

Satellite cells, required for muscle regeneration^14–17^, are typically quiescent and fuse into the multinucleated myotubes of skeletal muscle to maintain the tissue or in response to injury^18,19^. Following muscle injury, satellite cells exit quiescence, proliferate and then differentiate to repair muscle while a small number of cells self-renewal to maintain the quiescent satellite cell population^18^. While satellite cell dysfunction contributes to a variety of diseases including muscular dystrophy, cancer cachexia and age-induced muscle wasting^20–24^, whether Down syndrome trisomy affects satellite cells and contributes to Down syndrome muscle phenotypes is unknown. Since skeletal muscle dysfunction associated with Down syndrome includes muscle weakness, early onset age-induced atrophy and overall diminished mobility, Down syndrome trisomy may impact satellite cell function^25–29^.

Here we analyze Ts65Dn mice, an established mouse model of Down syndrome, that are trisomic for ~55% of the orthologous protein coding genes on human chromosome 21 and recapitulate many phenotypes observed in individuals with Down syndrome^30,31^. While pre-injury satellite cell numbers are normal, muscle regeneration is impaired in Ts65Dn mice because of a reduction in satellite cell expansion, arising from an inability of Ts65Dn satellite cells to complete their first cell division upon exit from quiescence. An accumulation of DNA damage and elevated levels of Usp16, a de-ubiquitinating enzyme whose gene is on chromosome 21, accompany the defects in Ts65Dn satellite cell division. The impairment of satellite cell function in Ts65Dn mice provides further evidence that stem cell dysfunction is a common contributor to multiple Down syndrome phenotypes.

## Results

### Impaired satellite cell function and muscle regeneration in Ts65Dn mice

**Figure 1:**
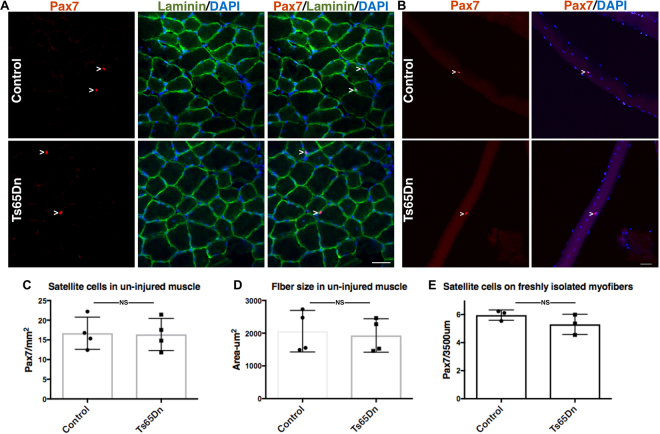
Satellite cell number and myofiber size are normal in un-injured Ts65Dn muscle. (**A**) Un-injured TA muscle sections stained with anti-Pax7 antibody to label satellite cells (red) and laminin (green) to label the basal lamina. Blue is DAPI. White carets mark satellite cells. (**B**) Myofibers isolated from EDL muscle were fixed immediately and stained with anti-Pax7 antibody to identify satellite cells. Blue is DAPI. White carets mark satellite cells. (**C**–**E**) Quantification of Pax7+ satellite cell number and average fiber size in Ts65Dn muscle compared to wild type (n = 3 or 4). Statistical significance was determined using Student’s t test using. P-value < 0.05 were considered significant. NS indicates not significant Scale bars are 40 μm.

Satellite cell number and myofiber size were analyzed in sections of un-injured tibialis anterior (TA) muscle from 5 mo old wild type mice and Ts65Dn mice by scoring for Pax7 immunoreactive satellite cells^15^ and by determining the myofiber cross-sectional area using laminin immunoreactivity to identify the myofiber basement membrane, respectively (Fig. 1A). No differences in either the numbers of Pax7+ satellite cells (Fig. 1A,C) or in the average myofiber cross-sectional area were observed between wild type TA muscles and Ts65Dn TA muscles (Fig. 1A,D). To confirm that satellite cell numbers between Ts65Dn muscles and wild type muscles were similar, Pax7+ satellite cell numbers were quantified on individual myofibers isolated from the extensor digitorum longus (EDL) muscle (Fig. 1B,E). Thus, no differences in average myofiber size or differences in the number of Pax7 expressing satellite cells were observed when comparing 5 mo old adult wild type muscles and Ts65Dn muscles.

**Figure 2:**
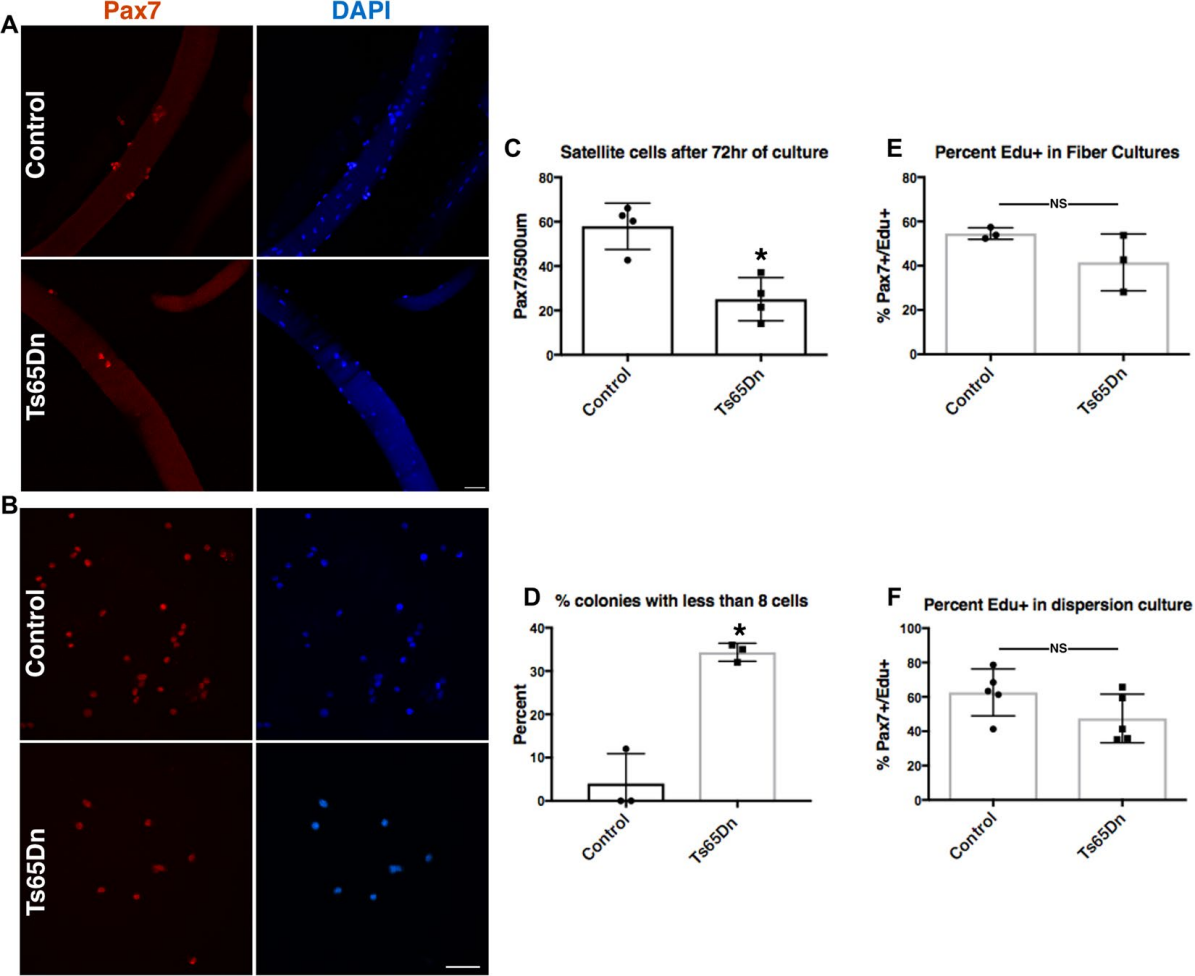
Impaired expansion of Ts65Dn satellite cells *in vitro*. (**A**) Pax7+ cells (red) on isolated EDL myofibers after 72 h of culture. Blue is DAPI. (**B**) Images of satellite cell colonies after 72 h of culture, immunoreactive for Pax7 (red) and DAPI (blue). (**C**) Quantification of satellite cell number normalized to myofiber length on myofibers cultured for 72 h (n = 4). (**D**) Quantification of colony size represented as percent of colonies with less than 8 cells after 72 h of culture (n = 3). (**E**) Quantification of EdU+ satellite cells on 72 h myofiber cultures where EdU was added to media 2 h prior to collection (n = 3). (**F**) Quantification of EdU+ cells in dispersion cultures after 72 h of culture and EdU given 2 h prior to collection (n = 5). Statistical significance was tested using Student’s t test. Asterisks indicates significant difference and P-value < 0.05. NS indicates not significant. Scale bars are 40 μm.

We compared the function of Ts65Dn satellite cells to wild type satellite cells by culturing satellite cells on individual myofibers isolated from EDL muscles^32,33^. Myofiber-associated satellite cells cultures were maintained for 72 h with the first satellite cell division occurring between 24–36 h and subsequent divisions occurring every 10–12 h thereafter^34^. Cultures were treated with EdU (5-ethynyl-2′-deoxyuridine**)** 2 h prior to collection to label cells in S-phase. A 57% reduction in Pax7 immunoreactive cell numbers occurred on myofibers cultured from Ts65Dn mice compared to myofibers from wild type mice (Fig. 2A,C). Clonal analysis of isolated Pax7+ satellite cells, cultured for 72 h, confirmed reduced expansion of Ts65Dn satellite cells, where 35% of satellite cell colonies in Ts65Dn cultures had 8 cells or less compared to only 5% of wild type colonies with 8 or fewer cells per colony (Fig. 2B,D). Although colony size was dramatically different between satellite cells isolated from Ts65Dn mice and wild type mice, the percent of EdU labeled cells did not differ between Ts65Dn and wild type cultures in either myofiber-associated satellite cell cultures or dispersed satellite cell cultures (Fig. 2E,F). Therefore, the deficient Ts65Dn Pax7+ satellite cell expansion is unlikely to arise from an overall slower cell cycle for Ts65Dn satellite cells compared to wild type satellite cells.

**Figure 3:**
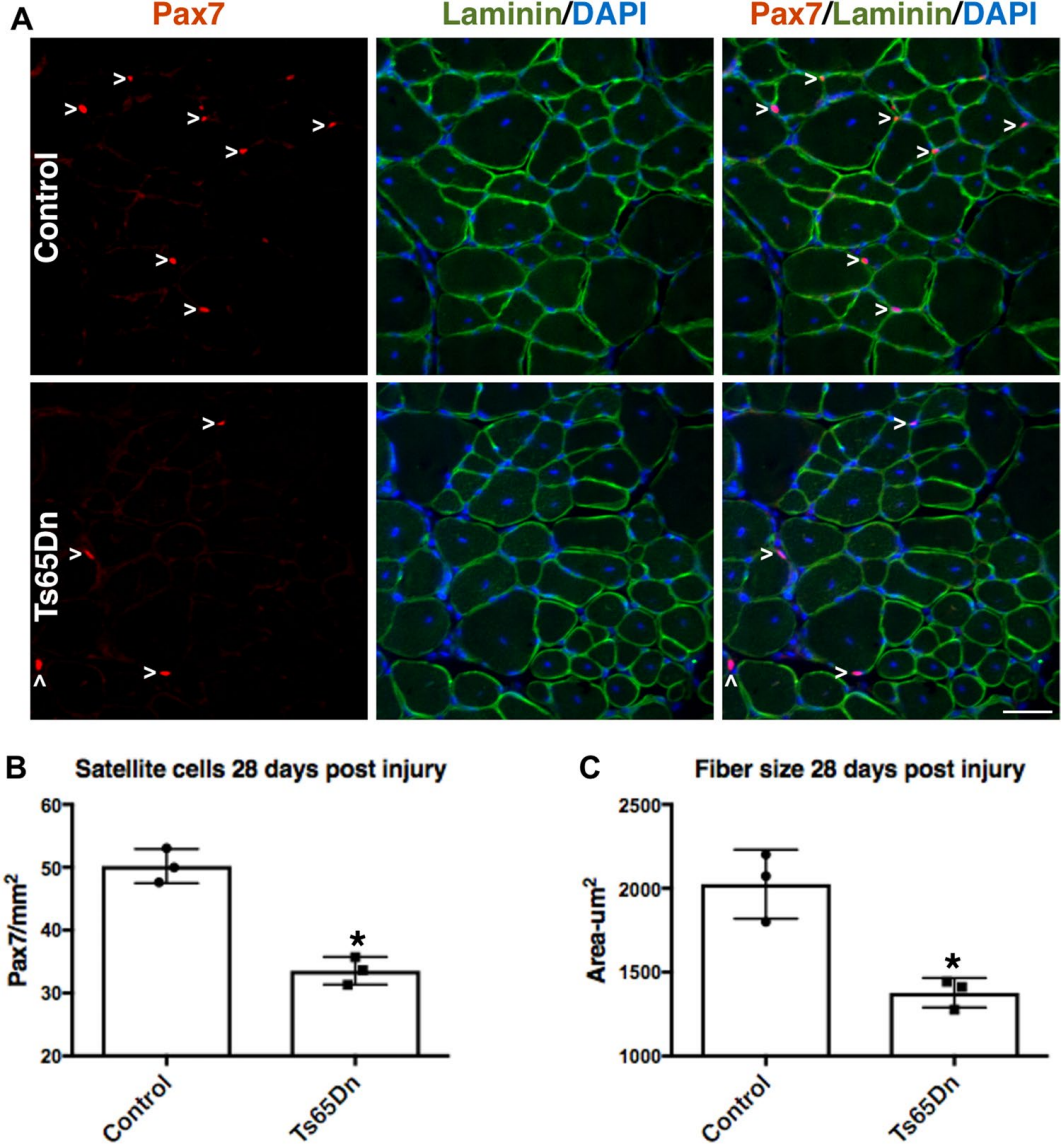
Impaired muscle regeneration in adult Ts65Dn mice. (**A**) TA muscle collected from 5mo old mice, 28 days post injury, immunoreactive for Pax7 (red) to identify satellite cells and immunoreactive for laminin (green) to identify the basal lamina. DAPI is blue. Carets mark satellite cells. (**B**–**C**) Quantification shows satellite cell number (**B**) and average fiber cross sectional area (**C**) in regenerated muscle tissue of wild type and Ts65Dn mice (n = 3). Statistical significance was tested using Student’s t test. Asterisks indicates significant difference and P-value < 0.05. Scale bar is 40 μm.

**Figure 4:**
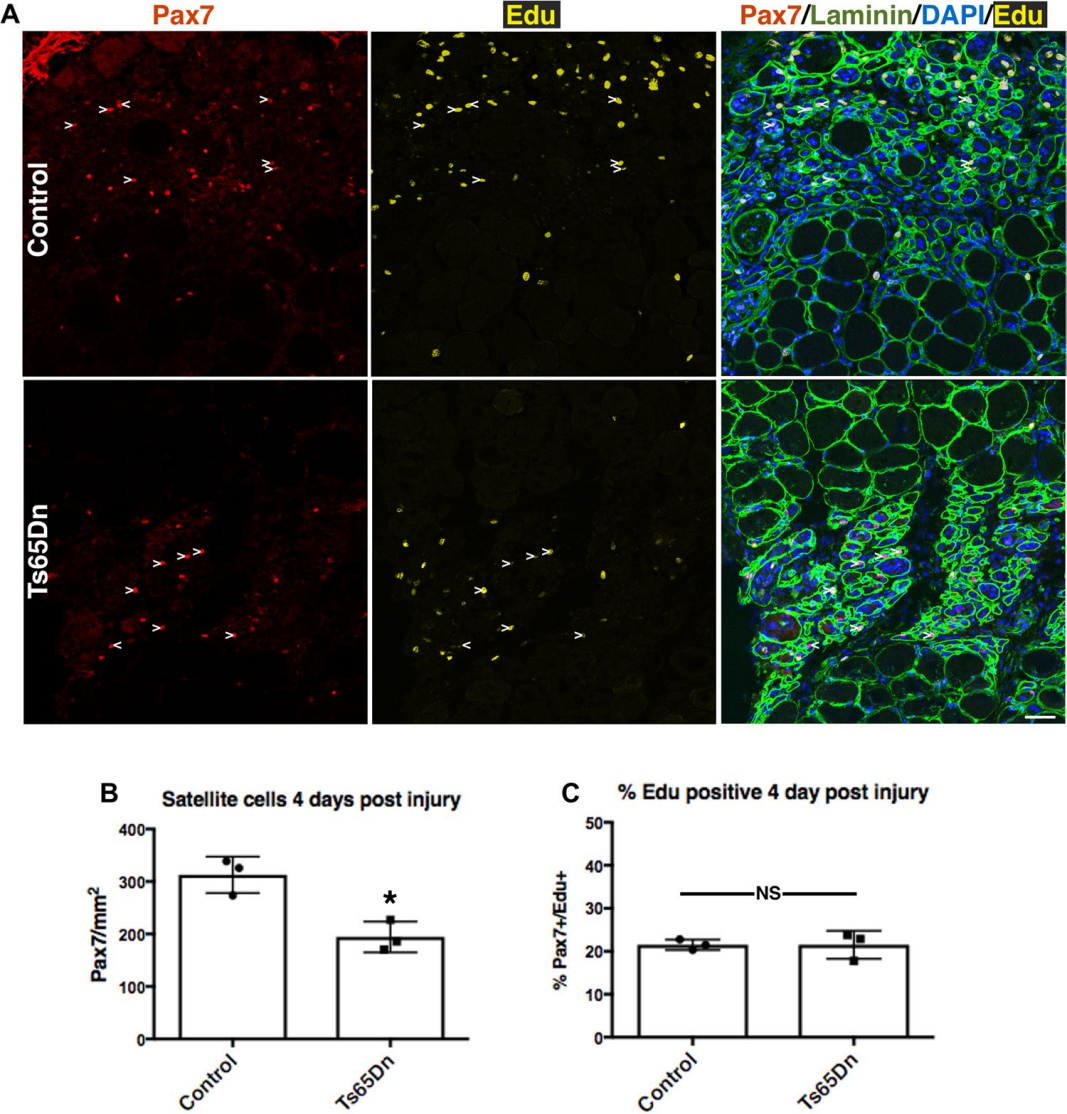
Reduced number of Ts65Dn satellite cells at 4 days post injury. (**A**) Images of TA muscle collected 4 days post injury were visualized for Pax7 immunoreactivity (red) to label satellite cells, laminin immunoreactiviey to mark myofibers (green) and EdU (yellow). EdU was injected 2 h prior to collection. Blue is DAPI. White carets mark EdU+ satellite cells. (**B**) Quantification of satellite cell numbers at 4 days post injury in Ts65Dn muscle and wild type muscle (n = 3). (**C**) Quantification showing the percentage of Pax7+/EdU+ cells in wild type TA muscle and Ts65Dn TA muscle at 4 day post injury (n = 3). Statistical significance was tested using Student’s t test. Asterisks indicates significant difference and P-value < 0.05. NS indicates not significant. Scale bar is 40 μm.

If the deficit in Ts65Dn satellite cell expansion is cell intrinsic and not an aberrancy arising from cell culture, then regeneration of Ts65Dn skeletal muscle should be impaired. Injection of the TA muscle with BaCl_2_ elicits a severe muscle injury with subsequent satellite cell expansion that peaks at 4 days post BaCl_2_ injury and resolves by 28 days post injury when satellite cells return to quiescence^16,35,36^. TA muscles from BaCl_2_-injected 5 mo old Ts65Dn mice and 5 mo old wild type mice were collected 28 days post BaCl_2_ injury and quantified for satellite cell numbers and myofiber size. The numbers of Pax7+ satellite cells were reduced by 1.5-fold in Ts65Dn muscle compared to wild type muscle (Fig. 3A,B), with a concomitant reduction in regenerated myofiber size (marked by the presence of central nuclei) in Ts65Dn muscle compared to wild type TA muscles (Fig. 3A,C). To examine satellite cell expansion *in vivo*, we injured the TA muscles of 5 mo old wild type and Ts65Dn mice using BaCl_2_ and injected EdU 2 h prior to tissue collection at 4 days post injury, the time point when satellite cells reach peak numbers^36^. Satellite cell expansion *in vivo* is impaired in Ts56Dn mice compared to wild type at 4d post-injury where reductions in Pax7 immunoreactive satellite cells are significant (Fig. 4A,B). No significant difference in the percentage of EdU positive Pax7+ satellite cells was observed between Ts65Dn and wild type TA muscles at 4d post injury (Fig. 4A,C). The impaired Pax7+ satellite cell expansion is likely responsible for the observed reductions in myofiber size in Ts65Dn mice compared to wild type mice following an induced muscle injury.

**Figure 5:**
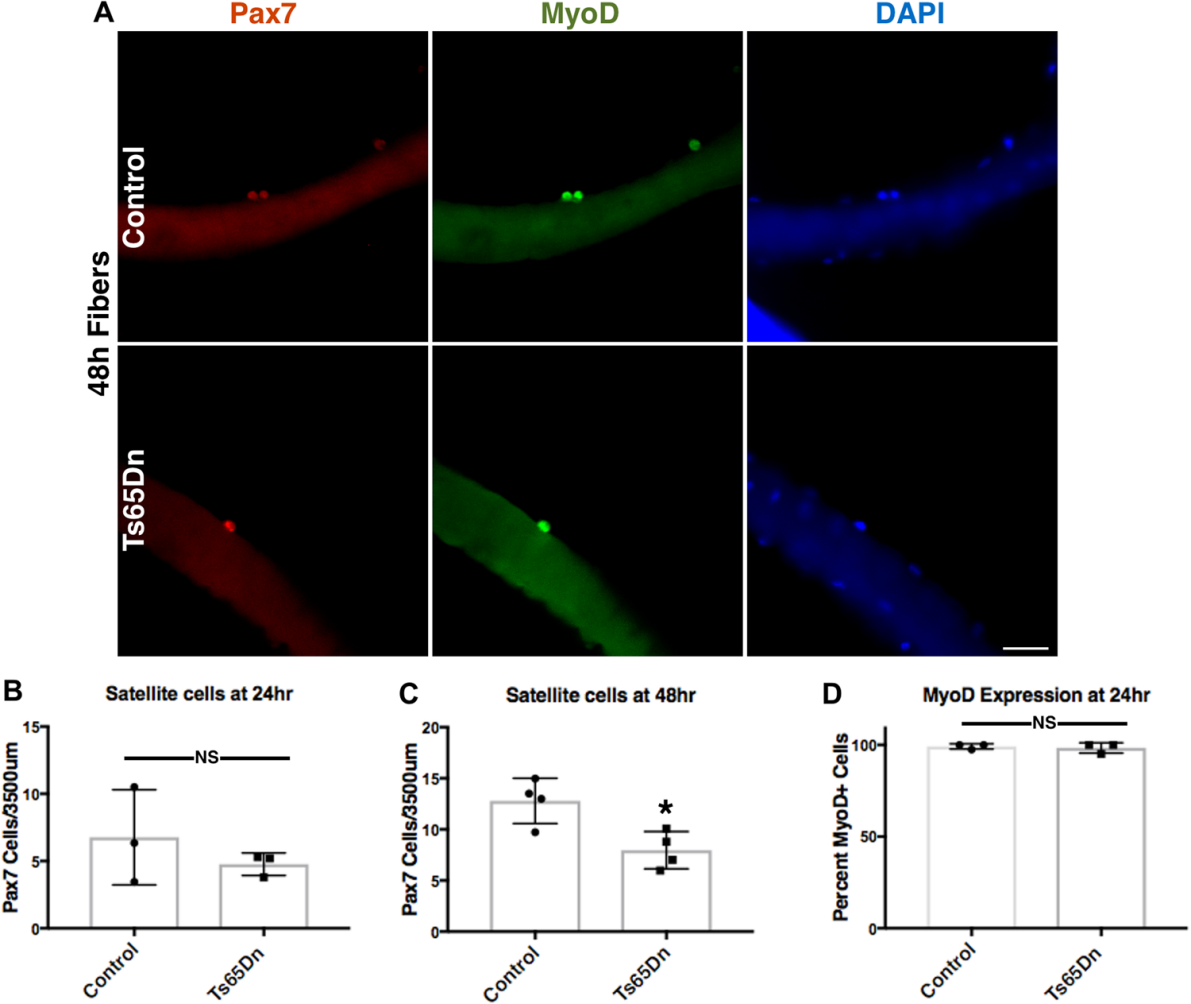
Ts65Dn satellite cells have an impaired first division following exit from quiescence. (**A**) Images of isolated myofibers grown in culture for 48 h and visualized for Pax7 immunoreactivity (red) and MyoD immunoreactivity (green). Blue is DAPI. Wild type satellite cells are seen as neighbor doublets while Ts65Dn satellite cells are predominately individual cells. (**B**) Quantification of satellite cell number normalized to myofiber length after 24 h for Ts65Dn myofibers and wild type myofibers (n = 3). (**C**) Quantification after 48 h of culture for shows a satellites cell on Ts65Dn myofibers compared to wild type myofibers (n = 4). (**D**) Quantification of MyoD positive satellite cells on myofibers cultured for 24 h from Ts65Dn mice and wild type mice (n = 3). Statistical significance was tested using Student’s t test. Asterisks indicates significant difference and P-value < 0.05. NS indicates not significant. Scale bar is 40 μm.

When satellite cells exit quiescence, Myf5 protein and MyoD protein are rapidly induced via inhibition of MyoD mRNA decay, permitting cell growth and mitosis between 24 h and 36 h after exit from G0^37,38^. Following the initial division, subsequent cell cycles are rapid with satellite cells dividing every 8 h–12 h. Isolation of myofibers and their associated satellite cells models *in vivo* injury, permitting analysis of cell cycle kinetics in culture^39–41^. No differences in the number of Pax7+ cells were observed 24 h post-isolation in myofiber-associated satellite cells from 5 mo old Ts65Dn mice compared to wild type mice (Fig. 5B). Moreover, the initial numbers of Pax7+ satellite cells on freshly isolated myofibers from Ts65Dn mice (5.30 Pax7+ satellite cells/myofiber length) were indistinguishable from Pax7+ satellite cell numbers on freshly isolated wild type myofibers (5.96 Pax7+ satellite cells/myofiber length). After 48 h of culture, Ts65Dn myofiber-associated Pax7+ satellite cell number are reduced compared to wild type (Fig. 5A,C), where most satellite cells on wild type myofibers appear as doublets, indicating a recent initial cell division (Fig. 5A). In contrast, the majority of myofiber-associated Pax7+ satellite cells on Ts65Dn myofibers have not yet completed their initial division after 48 h of culture (Fig. 5A). MyoD protein, required for S-phase entry, appears 3 h to 6 h post satellite cell activation and can be used to indicate G0 exit^38^. No differences in the percentage of MyoD+/Pax7+ satellite cells were observed in either wild type myofiber cultures or Ts65Dn myofiber cultures at 24 h post-isolation (Fig. 5A,D). Since both wild type satellite cells and Ts65Dn satellite cells exit G0 by 24 h in culture but the number of satellite cells on Ts65Dn myofibers fails to increase by 48 h of culture, the failure of Ts65Dn Pax7+ satellite cell numbers to increase most likely arises from a delayed or incomplete initial cell division following G0 exit.

### Ts65Dn satellite cells accumulate DNA damage and further functionally decline with age

**Figure 6:**
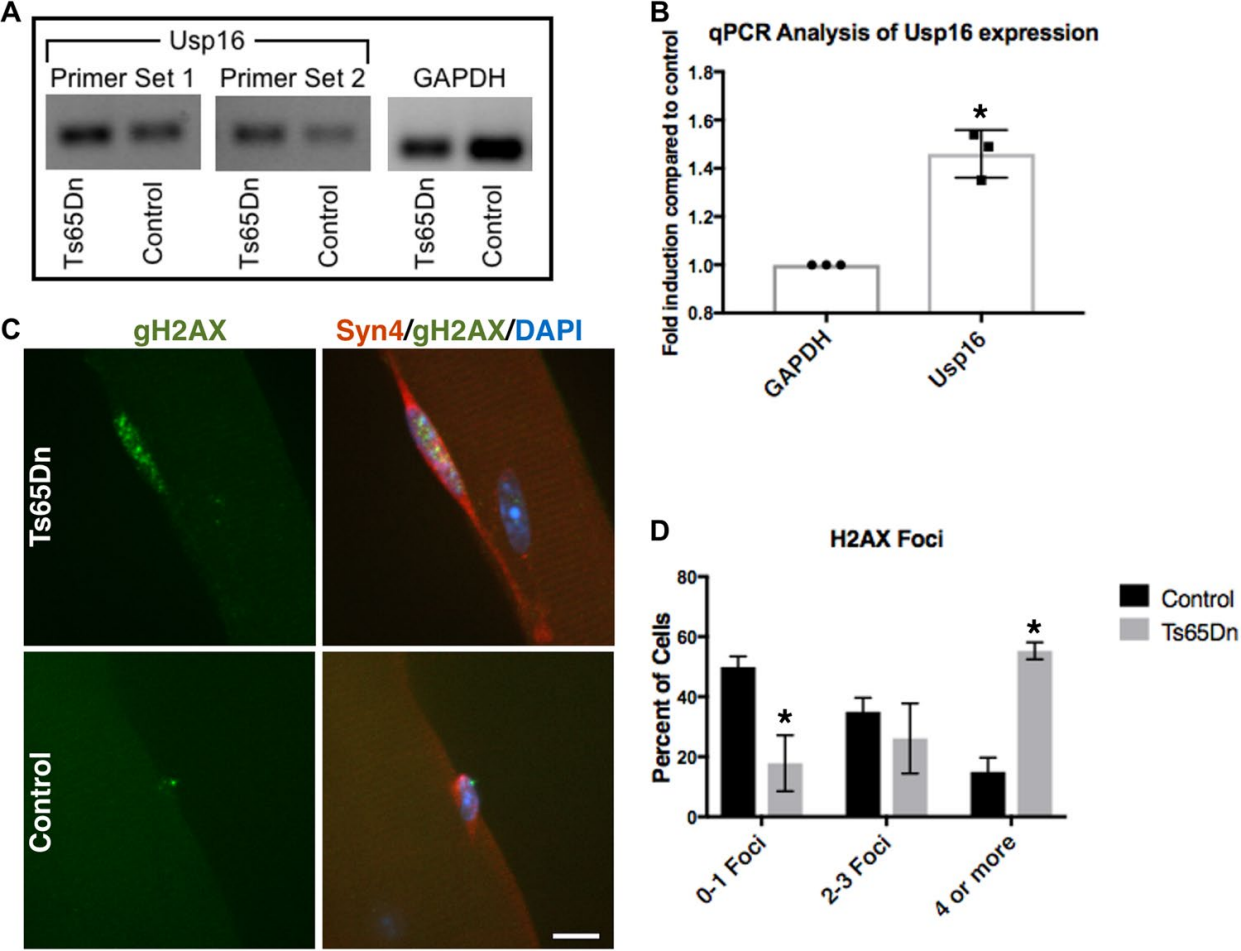
Increased Usp16 expression and DNA damage in Ts65Dn satellite cells. (**A**) Semi-quantitative RT-PCR analysis for Usp16 amplification products from cDNA of Ts65Dn satellite cells compared to wild type satellite cells. The images shown were cropped to save space (full-length gel included in Supplementary Information). (**B**) Quantitative real-time PCR for Usp16 expression in Ts65Dn satellite cells compared to wild type satellite cells (n = 3). (**C**) Images for isolated myofibers cultured for 24 h immunoreactive for γH2AX (green, DNA damage) and immunoreactive for Syndecan-4 (red, satellite cells). Blue is DAPI. (**D**) Quantification of DNA damage foci identified by γH2AX labeling in Ts65Dn satellite cells and wild type satellite cells (n = 3). Statistical significance was tested using Student’s t test. Asterisks indicates significant difference and P-value < 0.05. Scale bar is 10 μm.

Usp16, encoded on human chromosome 21, regulates gene expression, DNA damage repair, and cell cycle progression by controlling the ubiquitination state of histone 2 A (H2A)^42–44^. Usp16 expression is elevated 1.5-fold in Ts65Dn satellite cells compared to wild type satellite cells (Fig. 6A,B), as predicted by the three Usp16 alleles present in Ts65Dn mice. Since elevated Usp16 expression contributes to somatic stem cell dysfunction in Down syndrome^10^ and Usp16 represses DNA damage responses^44,45^, we asked if Ts65Dn satellite cells accumulate more DNA damage that wild type satellite cells. Individual myofibers were isolated from 6 mo old wild type mice as well as Ts65Dn mice and myofiber-associated satellite cells were cultured for 24 h. Staining for γH2AX, a variant of H2A that is rapidly phosphorylated at sites of double stranded DNA breaks^44–46^, revealed a four-fold increase of γH2AX foci in Ts65Dn satellite cells compared to wild type satellite cells (Fig. 6C,D). The increased DNA damage in Ts65Dn satellite cells may underlie their inability to effectively complete their first division upon exit from G0.

**Figure 7:**
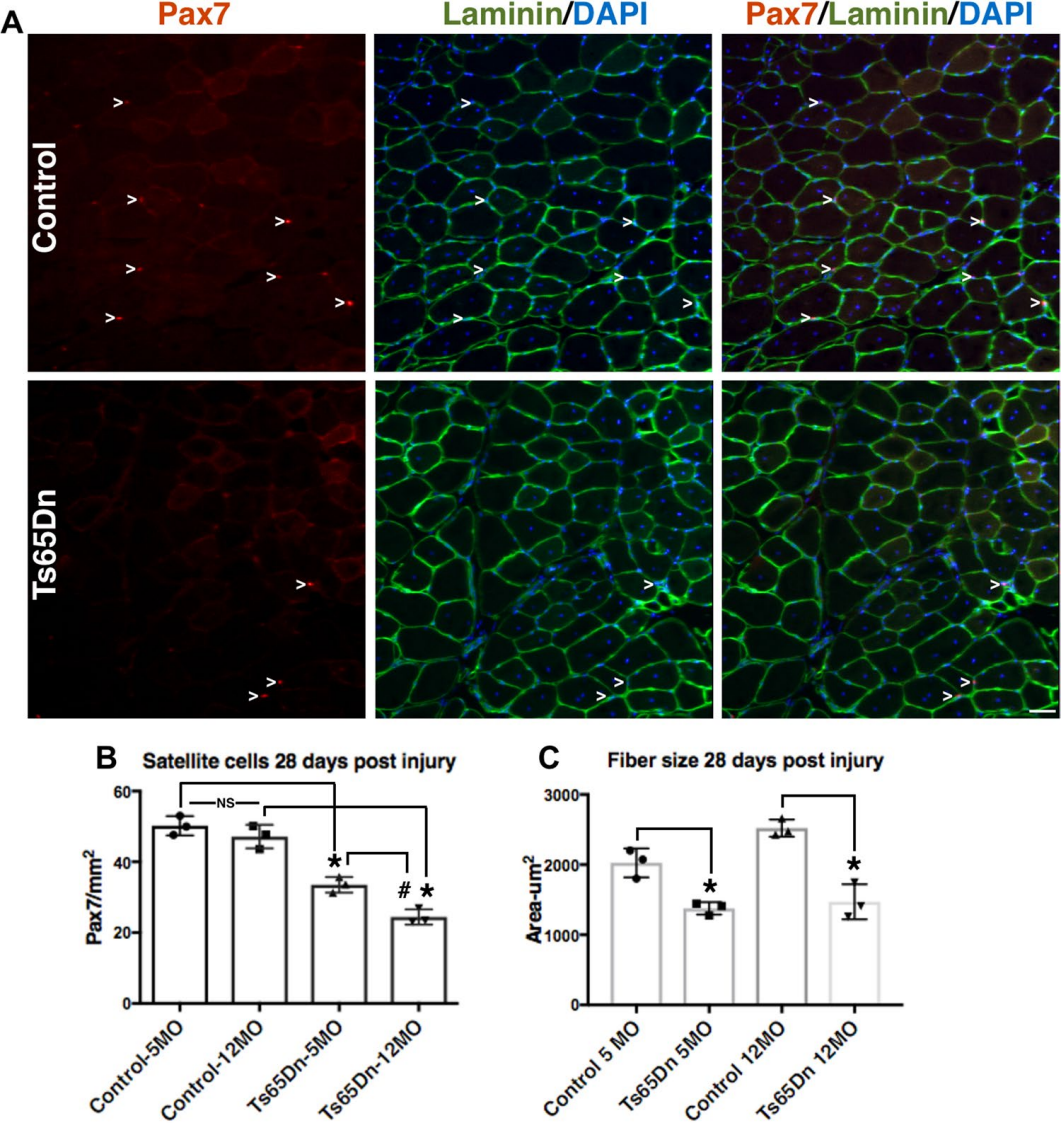
Further impairment of satellite cell function in 12 month old Ts65Dn tissue. (**A**) Satellite cells from TA muscles collected 28 days post injury from 12 mo old mice were quantified by Pax7 immunoreactivity (red) to mark satellite cells and laminin immunoreactivity (green) to identify the basal lamina. DAPI = blue. Carets mark satellite cells. (**B**) Satellite cell numbers quantified at 28 days post injury muscle for Ts65Dn regenerated muscle compared to wild type muscle at 5 mo of age and 12 mo of age. (**C**) Myofiber cross sectional area was measured in Ts65Dn regenerated muscle and wild type regenerated muscle at 5 mo of age and 12 mo of age. Statistical significance was tested using Student’s t test. Asterisks or pound sign indicates significant difference and P-value < 0.05. NS indicates not significant. Scale bar is 40 μm.

If DNA damage in Ts65Dn satellite cells accumulates over time, Ts65Dn satellite cell function maybe more severely impaired by age than wild type satellite cells. In wild type mice, age-related impairment of muscle regeneration does not appear until 18–20 mo of age and progressively worsens by 28–32 mo of age^20,21,47^. Muscle regeneration is impaired in 5 mo old and 12 mo old Ts56Dn mice compared to wild type mice (Fig. 7A–C). The average size of regenerated myofibers was reduced in Ts65Dn TA muscle compared to regenerated wild type TA muscles at 5 mo of age and 12 mo of age (Fig. 7A,C). However, myofiber size in injured TA muscles did not differ between 5 mo old and 12 mo old Ts65Dn mice (Fig. 7C). The numbers of Pax7+ satellite cells in Ts65Dn regenerated muscle declines from 5 mo to 12 mo of age and is significantly reduced compared to the numbers of wild type Pax7+ satellite cells in injured muscles at both 5 mo and 12 mo of age (Fig. 7A,B). The numbers of Pax7+ satellite cells in un-injured 12 mo old wild type TA muscles was not significantly different than those in 12 mo old Ts65Dn TA muscles (17.95 ± 2.28 Pax7+ satellite cells/mm^2^ in wild type vs. 15.55 ± 1.14 Pax7+ satellite cells/mm^2^ in Ts65Dn). The continuing decline in satellite cell numbers following regeneration in Ts65Dn muscle that occurs with age is consistent with an advancing decline in satellite cell function caused by accumulation of DNA damage.

## Discussion

Here we report that reduced satellite cell expansion impairs skeletal muscle regeneration in Ts65Dn mice. Satellite cell expansion defects in Ts65Dn mice arise from an impaired initial cell division following exit from quiescence. However, there are no differences in either cell cycle rates or the time to exit quiescence between Ts65Dn satellite cells and wild type satellite cells. Ts65Dn satellite cells over express the de-ubiquitinating enzyme Usp16, accumulate double stranded DNA breaks, and lose regenerative capability when compared to wild type satellite cells. Therefore, Ts65Dn satellite cell dysfunction may contribute to multiple Down syndrome muscle phenotypes providing additional evidence that somatic stem cell deficiencies contribute to Down syndrome phenotypes.

Down syndrome phenotypes associated with skeletal muscle and mobility include muscle weakness, low muscle tone, and fragile joints^48–51^. Satellite cells maintain skeletal muscle, are essential for skeletal muscle repair^19,52^, and thus, decreases in muscle strength in individuals with Down syndrome may arise from deficits in satellite cell function^48–50^. Although individuals with Down syndrome benefit from physical training, and can increase strength with resistance exercise, it is unclear if their exercise response is blunted^27,29,53^. Since satellite cells respond to exercise^54–56^, determining if satellite cell dysfunction contributes to muscle weakness or impairs the exercise response in individuals with Down syndrome may be clinically relevant.

Our data add skeletal muscle stem cells to a growing list of dysfunctional stem cells and dysfunctional progenitor cells in Down syndrome that include HSCs, neuronal stem cells, mammary epithelial cells and neural crest progenitors^8,10,12,57,58^. Common functional stem cell defects associated with Down syndrome include impaired expansion and impaired self-renewal^10,11^, which may contribute to accelerated aging phenotypes^10,12,13,59^. Declines in Ts65Dn satellite cell function, which are apparent in 5 mo old Ts65Dn mice and are exacerbated in 1 year old Ts65Dn mice do not occur in1 year old wild type mice. The declines in Ts65Dn satellite cell function correlate with sarcopenia appearing at younger ages with more pronounced atrophy in Ts65Dn mice than in age-matched wild type mice^28^. Bolstering somatic stem cell function in individuals with Down syndrome may slow the accelerated aging and thus, is a potential therapeutic target.

What molecular mechanisms drive Down syndrome stem cell and satellite cell dysfunction? In hematopoietic stem cells, Usp16 over expression prematurely induces senescence via p16^Ink4a^ expression^10^. In Ts65Dn satellite cells, Usp16 expression is increased but we found no evidence of increased p16^Ink4a^ expression (data not shown). However, the presence of γH2AX foci and increased Usp16 expression in Ts65Dn satellite cells could be responsible for increased DNA damage and decreased DNA damage repair^60^. Ubiquintation of histone 2A at sites of DNA damage recruits DNA damage repair proteins, while Usp16 and other de-ubiquintases restore ubiquitin levels to normal approximately 24 h after damage^60–62^. Thus, the increased Usp16 expression in TS65Dn satellite cells could disrupt DNA repair, induce DNA damage, and impair satellite cell expansion. Over expression of Usp16 is likely not solely responsible for the increased DNA damage as Down syndrome cells have an increased susceptibility to genotoxic stress^63^. Moreover, increased oxidative stress in Down syndrome cells leads to increased DNA damage and to increased apoptosis^64–67^. While we could not detect apoptotic cells on Ts65Dn myofiber cultures using TUNEL staining (data not shown), it is possible that a small number of satellite cells are undergoing apoptosis. Although, our EdU analysis did not detect differences in cell cycle rates for Ts65Dn satellite cells compared to wild type cells, cell cycle deficiencies have been reported in other Ts65Dn adult stem cell populations^10,11^. Thus, we cannot rule out cell cycle defects not detectable in our assay. Our data suggest that the primary mechanism responsible for reducing Ts65DN satellite cell numbers is a delay in the initial cell division, reducing final cell numbers. The cause of DNA damage in Ts65Dn satellite cells and how DNA damage leads to reductions in satellite cell numbers remains unknown. Whether the reduced satellite cell numbers arise from cell autonomous changes has not been addressed and cell non-autonomous effects may contribute to satellite cell dysfunction. Furthermore, the Ts65Dn muscle regeneration defects are not likely caused by satellite cell defects alone. For example, immune deficiency observed in Down syndrome^68,69^ could contribute to satellite cell dysfunction, as immune cells are required for skeletal muscle regeneration^70–73^. In conclusion, the data presented here identify potential causes of Down syndrome associated skeletal muscle weakness. In addition, satellite cells are another somatic stem cell that function aberrantly in a mouse model of Down syndrome and our data link DNA damage to Down syndrome stem cell dysfunction.

## Methods

### Mice

Mice were bred and housed according to National Institutes of Health (NIH) guidelines for the ethical treatment of animals in a pathogen-free facility at the University of Colorado at Boulder. University of Colorado Institutional Animal Care and Use Committee (IACUC) approved all animal protocols and procedures. Ts65Dn mice were purchased from Jackson Labs and Ts65Dn trisomic female mice (Jackson Labs Stock No. 005252) were crossed with wild type disomic F1 hybrid male mice (Jackson Laboratory Stock No. 001875). The male F1 hybrid mice are offspring of a cross between C57BL/6JEiJ females (B6Ei) and C3H/HeSnJ males (C3Sn) and commonly used in crosses as a background for deleterious mutations. For all experiments, wild type mice are disomic sex-matched littermates from the crosses described above.

### Mouse Injuries and EdU injections

Mice at either 5 mo of age or 12 mo of age were anesthetized with 3% isofluorane and the left TA muscle was injected with 50 μL of 1.2% BaCl_2_. Intraperitoneal (IP) injections of 10 mM EdU (Carbosynth), resuspended in water, were given 2 h prior to collection at a volume of 100 µl/25 g mouse weight.

### Immunofluorescence staining of tissue section

The TA muscle was dissected, fixed for 2 h on ice cold 4% paraformaldehyde, and then transferred to PBS with 30% sucrose at 4 °C overnight. Muscle was mounted in O.C.T. (Tissue-Tek®) and cryo-sectioning was performed on a Leica cryostat to generate 8 μm sections. Tissues and sections were stored at −80 °C until staining. Tissue sections were post-fixed in 4% paraformaldehyde for 10 min at room temperature (RT) and washed three times for 5 min in PBS. For heat-induced epitope retrieval, required for anti-Pax7 antibody staining, post-fixed slides were placed in citrate buffer, pH 6.0, and subjected to 6 min of high pressure-cooking in a Cuisinart model CPC-600 pressure cooker set on high pressure. For immunostaining, tissue sections were permeabilized with 0.25% Triton-X100 (Sigma) in PBS containing 3% bovine serum albumin (Sigma) for 45 min at RT, incubated with primary antibody at 4 °C overnight followed by incubation with a secondary antibody at RT for 1 h. Primary antibodies included mouse anti-Pax7 (DSHB) at 1:1000 and rabbit anti-laminin (Sigma-Aldrich) at 1:200. Alexa-488, 555, and 647 secondary antibodies (Molecular Probes) were used at a 1:750 dilution. For analysis that included EdU detection, EdU staining was completed prior to antibody staining using the Click-iT EdU Alexa fluor 488 detection kit (Molecular Probes) following the manufacturer’s protocols. Sections were incubated with 1 μg/mL DAPI for 10 min at room temperature then mounted in Mowiol supplemented with DABCO (Sigma-Aldrich) as an anti-fade agent.

### Analysis of tissue sections

For analysis of immunostained TA muscle sections in Figs 1, 3, 4 and 7 one N is a biological replicate comparing a TA muscle from a Ts65Dn mouse with an aged-matched and sex-matched wild type mouse (disomic littermates). For biological replicates within a figure, both male and female mice were used. The sex of the mice used for the TA analysis are as follows: Fig. 1 (2 females and 1 male), Fig. 3 (1 female and 2 males), Fig. 4 (2 females and 1 male), Fig. 7 (3 males). Uninjured TA muscles are from the contralateral hindlimbs of BaCl_2_-injured mice in Fig. 1. For Pax7 cell quantification of TA muscle sections a minimum of eight images were scored from a minimum of 5 sections for each N and normalized to area. Images were blinded for quantification. When injured tissue was analyzed, Pax7+ cells were quantified only in areas of injury as identified by myofibers containing centrally located nuclei, scored and normalized as described for uninjured muscle. A minimum of 5 images from 5 sections were scored for myofiber size where images were blinded. In injured muscle, only myofibers with central nuclei were scored for size. For each N a minimum of 400 myofibers were scored.

### Myofiber isolation, immunostaining and culture

The extensor digitorum longus (EDL) muscles were dissected, placed into 400 U/mL collagenase at 37 °C for 1.5 h with shaking and then placed into Ham’s F-12C supplemented with 15% horse serum to inactivate the collagenase. Individual EDL myofibers were separated and isolated using a glass pipet. For immediate analysis, myofibers were fixed in 4% paraformaldehyde for 10 min and stored in PBS for immunostaining. Intact myofibers were cultured in Ham’s F-12C media supplemented with 15% horse serum and 0.5 nM FGF-2 at 6% O_2_. Where assayed, EdU was added directly to the media at a final concentration of 10 μm. Myofibers were permeabilized with 0.25% Triton-X100 in PBS containing 3% bovine serum albumin (Sigma) for 45 min at RT and incubated with primary antibody at 4 °C overnight followed by incubation with secondary antibodies at room temperature for 1 h. Primary antibodies used were mouse anti-Pax7 (DSHB at 1:1000, rabbit anti-MyoD (Santa Cruz) at 1:1000, chicken anti-Syndecan4 at 1:1000, and mouse anti-phospho-H2A.X (EMD-Millipore 05–636) at 1:250. Alexa-488, 555 and 647 secondary antibodies (Molecular Probes) were used at a 1:750 dilution. For analysis that included EdU detection, EdU staining was completed prior to antibody staining using the Click-iT EdU Alexa fluor 488 detection kit (Molecular Probes) following the manufacturer’s protocol. Following immunostaining, myofibers were incubated with 1 μg/mL DAPI for 10 min at RT then mounted in Mowiol supplemented with DABCO (Sigma-Aldrich) as an anti-fade agent.

### Analysis of myofibers and myofiber cultures

Satellite cells for each N of Figs 1, 2, 5, 6 were scored from EDL myofibers isolated from separate Ts65DN and aged-matched and sex-matched wild type mice (each N are EDL myofibers from different mice). For biological replicates within a figure male and female mice were used. The sex of the mice used are: Fig. 1 (2 females and 1 male), Fig. 2 (2 females and 2 males), Fig. 5 (2 males and 2 females), Fig. 6 (2 females and 1 male). A minimum of 25 immunostained myofibers mounted on slides were scored blinded for Pax7 immunoreactivity. A minimum of 40 immunostained satellite cell images were scored blinded for ɣH2AX foci.

### Dispersion cultures

Hindlimb muscles were dissected, minced, and digested in 400 U/mL collagenase type I in Ham’s F-12C at 37 °C for 1 h with gentle vortexing every 10 min. Digested muscle was placed into Ham’s F-12C supplemented with 15% horse serum to inactivate the collagenase and passed successively through three cell strainers: 100 μm, 70 μm, and 40 μm (BD Falcon). The flow through was centrifuged at 1500x*g* for 5 min and the cell pellets re-suspended in Ham’s F-12C supplemented with 15% horse serum and 0.5 nM FGF-2. The resuspended cells were pre-plated for 2 hours on plastic tissue culture plates to remove adherent fibroblasts and then the remaining suspended cells plated onto coverslips coated with 0.66% gelatin or onto 10 cm gelatin-coated tissue culture dishes (colonies were ≥90% satellite cells). Clonal analysis was performed with cells plated at ~100 cells/single well of a 6 well tissue culture plate containing a coverslip to permit identification of individual colonies. When required, EdU was added directly to the media for a final concentration of 10 μm. Satellite cell explant cultures were fixed and immunostained as described for myofibers.

### Analysis of dispersion cultures

Each N is a biological replicate from an individual mouse where muscle was isolated from either a Ts65DN mouse or an aged-matched and sex matched-wild type mouse (disomic littermate). For biological replicates within a figure, both male and female mice were used. The sex of the mice used were: Fig. 2 (3 females and 2 males), Fig. 6 (1 female and 2 males). Scoring of coverslips was performed blinded where a minimum of 100 satellite cells or a minimum of 20 colonies for clonal analysis per N were scored, respectively.

### Semi quantitative RT-PCR and real time qPCR

RNA was isolated from dispersion culture cells after 72 h of growth using the RNeasy kit (Qiagen) following manufacturers protocol. The Superscript III first strand synthesis system (ThermoFisher) was used for cDNA synthesis from 250 ng isolated RNA. Transcript expression analysis using standard semi-quantitative PCR analysis (FastStart Taq DNA polymerase, Sigma-Aldrich) at 25 cycles was done using a C1000 Touch Thermocycler (Bio-Rad). For real time quantitative PCR, iQ SYBR green supermix (BioRad) was used with a CFX384 Touch Real-Time Thermocycler (Bio-Rad).

Primer sequences: Usp16 For1-ctacacgacaccgagatccg, Usp16 Rev1-tgaagttcttacgccagcttg, Usp16 For2-gctggcgtaagaacttcaaaac, Usp16 Rev2-agtcctttcacagttatctggga, GAPDH For aggtcggtgtgaacggatttg, GAPDH Rev ggggtcgttgatggcaaca.

### Microscopy and image analysis

All images were captured on a Nikon inverted spinning disk confocal microscope or Olympus IX81 Inverted wide-field microscope. Objectives used on the Nikon were: 10x/o.45NA Plan Apo, 20x/0.75NA Plan Apo and 40x/0.95 Plan Apo. Objectives used on the Olympus were 10x/0.40NA and 20x/0.70NA. Images were processed using Fiji ImageJ. Confocal stacks were projected as maximum intensity images for each channel and merged into a single image. Brightness and contrast were adjusted for the entire image as necessary. Both satellite cell numbers and average myofiber size were scored manually using Fiji ImageJ on blinded images.

### Statistics

All statistical analyses were performed in Prism (GraphPad). To assess statistical significance, two-tailed, unpaired Student’s *t* test were performed and *p* < 0.05 was considered significant. At least three different animals per genotype and 3 animals per age group were used in all experiments.

## Electronic supplementary material

Supplementary Info
