# Supplementary material for: Muscle stem cell dysfunction impairs muscle regeneration in a mouse model of Down syndrome
Source: Sci Rep. 2018 Mar 9;8:4309. doi: 10.1038/s41598-018-22342-5 (PMC5844921; doi:10.1038/s41598-018-22342-5)
Supplement: Supplementary file 1 — Supplementary Info [file 41598_2018_22342_MOESM1_ESM.pdf]

Muscle stem cell dysfunction impairs muscle regeneration in a mouse model of Down syndrome

Bradley Pawlikowski[1,2], Nicole Dalla Betta[1,2], Tiffany Antwine[1,2], Darian Williams[1,2], Bradley Olwin[1,2,*]

[1] Department of Molecular, Cellular and Developmental Biology, University of Colorado, Boulder CO 80039

[2] Linda Crnic Institute for Down Syndrome, University of Colorado School of Medicine, Aurora, United States.

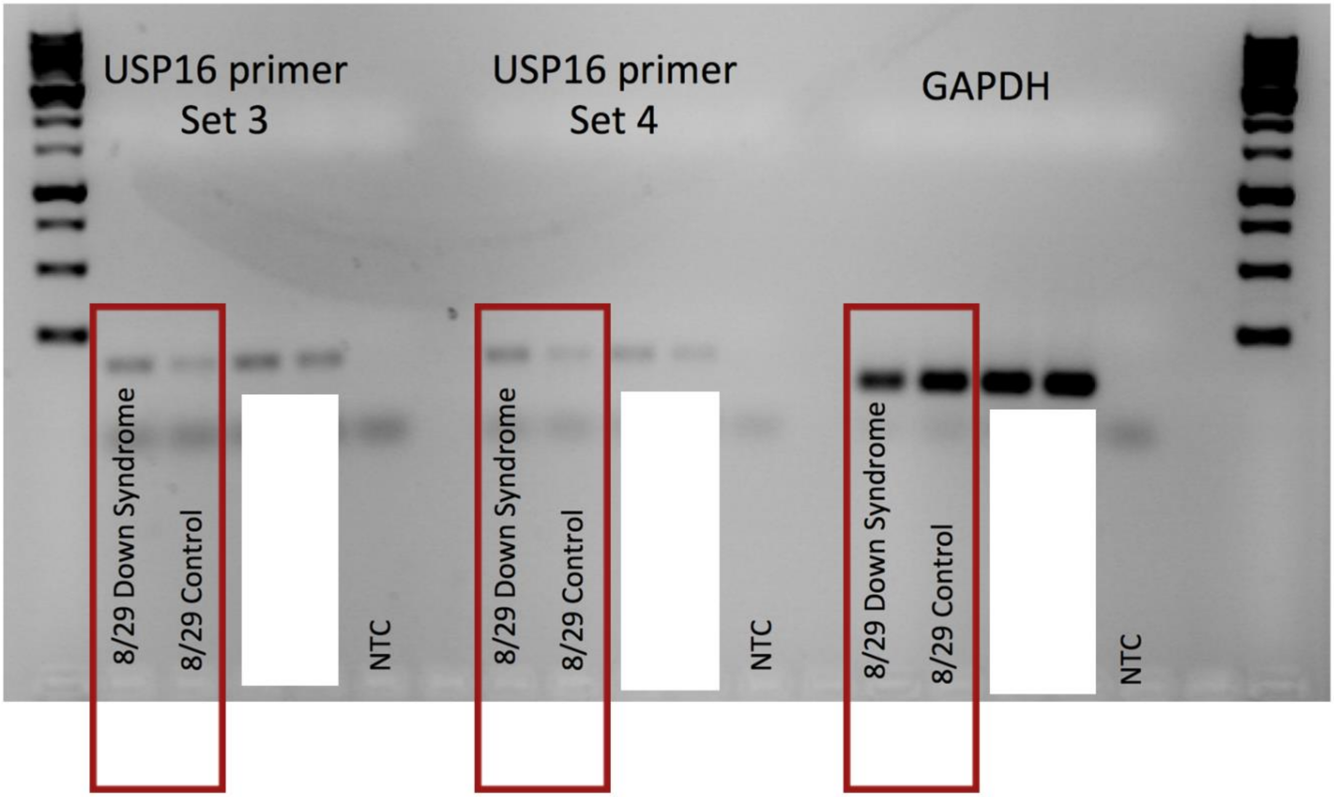

Supplemental Figure S1. Image of the full length gel shown as cropped images in Fig. 6A. The red boxes indicate the data presented in Fig. 6A. The data outside the red boxes are not presented or discussed in this manuscript.
